# Retraction of: PHTF2 regulates lipids metabolism in gastric cancer

**DOI:** 10.18632/aging.205842

**Published:** 2024-04-30

**Authors:** Yuhua Chi, Haiyan Wang, Fengsong Wang, Mingcui Ding

**Affiliations:** 1 Department of Oncology, People’s Hospital of Rizhao, Rizhao 276800, Shandong Province, China

**Keywords:** PHTF2, lipids metabolism, tumorigenesis, gastric cancer

**This article has been retracted:** Aging has completed its investigation of this paper. We found that the immunohistochemistry images in **Figure 2H**, showing PHTF2 expression pattern in gastric cancer tissues, were previously published in an unrelated paper from a different lab studying PBX1 protein in clear cell renal carcinoma [1]. We also found that the image of immunofluorescence staining in **Figure 3I**, representing the neutral lipids content detected by double staining with BODIPY 493/503 dye in GCIY/shACSL1 gastric cancer cells, is a duplication of a BODIPY image of Zebrafish liver cells from an unrelated paper [2]. In addition, the colony formation images shown in **Figure 4B** are the same as in an earlier published paper from an unrelated team of authors studying bladder cancer [3]. The authors were informed about the results of the Aging investigation and all authors agreed that the article should be retracted.
